# Functional Integration of Human Neural Precursor Cells in Mouse Cortex

**DOI:** 10.1371/journal.pone.0120281

**Published:** 2015-03-12

**Authors:** Fu-Wen Zhou, Jeff M. Fortin, Huan-Xin Chen, Hildabelis Martinez-Diaz, Lung-Ji Chang, Brent A. Reynolds, Steven N. Roper

**Affiliations:** 1 Department of Neurosurgery and the McKnight Brain Institute, University of Florida, Gainesville, Florida, United States of America; 2 Department of Molecular Genetics and Microbiology, University of Florida, Gainesville, Florida, United States of America; University of Utah, UNITED STATES

## Abstract

This study investigates the electrophysiological properties and functional integration of different phenotypes of transplanted human neural precursor cells (hNPCs) in immunodeficient NSG mice. Postnatal day 2 mice received unilateral injections of 100,000 GFP+ hNPCs into the right parietal cortex. Eight weeks after transplantation, 1.21% of transplanted hNPCs survived. In these hNPCs, parvalbumin (PV)-, calretinin (CR)-, somatostatin (SS)-positive inhibitory interneurons and excitatory pyramidal neurons were confirmed electrophysiologically and histologically. All GFP+ hNPCs were immunoreactive with anti-human specific nuclear protein. The proportions of PV-, CR-, and SS-positive cells among GFP+ cells were 35.5%, 15.7%, and 17.1%, respectively; around 15% of GFP+ cells were identified as pyramidal neurons. Those electrophysiologically and histological identified GFP+ hNPCs were shown to fire action potentials with the appropriate firing patterns for different classes of neurons and to display spontaneous excitatory and inhibitory postsynaptic currents (sEPSCs and sIPSCs). The amplitude, frequency and kinetic properties of sEPSCs and sIPSCs in different types of hNPCs were comparable to host cells of the same type. In conclusion, GFP+ hNPCs produce neurons that are competent to integrate functionally into host neocortical neuronal networks. This provides promising data on the potential for hNPCs to serve as therapeutic agents in neurological diseases with abnormal neuronal circuitry such as epilepsy.

## Introduction

Proper brain function requires a strict balance between neuronal excitation and inhibition [1–2]. Reduced inhibition (e.g., due to loss of inhibitory interneurons) in neuronal networks can lead to neurological disorders including epilepsy [3–6]. Cell-based therapy to replace lost or malfunctioning inhibitory interneurons has been hailed as a potential biologic therapeutic for these disorders [6–10]. Previous studies have demonstrated that neural stem and progenitor cells from animal embryos and fetuses possess the capacity to differentiate into GABAergic interneurons that form functional synaptic connections and integrate into the host brain circuitry when transplanted into animals [11–12]. Transplanted human embryonic and fetal stem cells in both younger and adult animals can develop into regionally appropriate neuron types including interneurons [13–20]. Transplantation of animal and human embryonic stem cells have shown promise in improving behavioral deficits in animal models of diseases including Parkinson’s disease, Huntington’s disease and epilepsy [8–9, 21–23] and promoting recovery after experimental spinal cord and brain injury [24–29], although it is not clear which neuronal type(s) contribute to the improvement. Previous studies have revealed that transplanted animal and human embryonic stem cell-derived GABAergic neuron precursors can attenuate behavioral deficits in rodent models of human disorders [2, 5, 7, 17, 23, 30–32]. Clinical benefit has been reported in some patients with human stem cell transplantation, such as Huntington's disease [33], amyotrophic lateral sclerosis [34] and Pelizaeus-Merzbacher Disease [35]. The major goal of human stem cell transplantation for neurodegenerative disorders is to elucidate its role in disease treatment. To achieve this goal it is essential to investigate both the specific phenotypes of transplanted stem cells and the ability of these cells to influence the behavior of the host neural circuitry in animal studies.

Transplanted animal stem and progenitor cells that can generate different types of neurons have been studied intensively. However, human stem cell transplantation has not been investigated to the same degree. This study investigated the electrophysiological and histological properties of different types of neurons derived from transplanted human neural precursor cells (hNPCs). In the neocortex, 70~80% of neurons are excitatory pyramidal neurons, and most of the others are GABAergic inhibitory interneurons [36]. GABAergic interneurons can be distinguished by their electrophysiology and expression of specific molecular markers [37]. GABAergic interneurons expressing the calcium-binding proteins, parvalbumin (PV) or calretinin (CR), or the neuropeptide, somatostatin (SS), comprise three separate families of interneurons, which account for the majority of neocortical GABAergic interneurons [37–38]. In the present study, we transplanted hNPCs into the neocortex of postnatal day 2 NOD.*Cg-Prkdc* ^*scid*^ *Il2rg* ^*tm1Wjl*^/SzJ (NSG) mice, an immunodeficient mouse, and determined the histological and electrophysiological properties of four types of neurons and the functional integration of grafted cells within host brain circuitries.

## Materials and Methods

### Animals

Pregnant NSG mice were purchased from the Jackson Laboratory (Bar Harbor, ME, USA). Postnatal day 2 (P2) mice received transplantation of hNPCs into the neocortex. Offspring were weaned on P21. Male or female offspring were used for electrophysiological and histological experiments at 8 weeks (8 W, P56–P61) after transplantation. All mice were maintained on 12 h light/dark cycles and were given *ad libitum* access to food and water.

### Ethics statement

All procedures were performed in accordance with guidelines approved by the National Institutes of Health and the Institutional Animal Care and Use Committee at the University of Florida.

### Culture of human neural precursor cells

Human NPCs were derived from the telencephalon of a single fetus after routine legal abortion at ten weeks of age, as previously published [39–41]. For transducing hNPCs, the lentiviral vector encoding eGFP was constructed under control of human EF1a enhancer/promoter in pTYF backbone, and lentivirus was generated as previously described [42]. The cells were seeded in a 12-well plate at 1×10^5^ cells per well one day before transduction, and then incubated with the lentivirus at approximately 5 moi (multiplicity of infection) supplemented with 8 μg/ml polybrene (Sigma) in culture medium overnight. Fresh medium was added the next day.

Cells were serially passaged using the non-adherent culturing technique called the Neurosphere Assay [43–45]. Briefly, the Neurosphere Assay entailed plating the cells as free-floating single cells at 100,000 cells/ml in NS-A medium (90% Neurocult NS-A Basal Medium Human plus 10% Human NeuroCult NS-A Proliferation Supplements, #05750 and 05753, respectively; StemCell Technologies, Vancouver, BC, Canada), supplemented with recombinant human epidermal growth factor (R&D Systems, Minneapolis, MN, USA) at a final concentration of 20 ng/ml, recombinant human basic fibroblast growth factor (R&D Systems) at a final concentration of 20 ng/ml, heparin (Sigma-Aldrich, St. Louis, MO, USA) at a final concentration of 0.7 USP units/ml, recombinant human leukemia inhibitory factor (Millipore, Darmstadt, Germany) at a final concentration of 10 ng/ml, and dehydroepiandrosterone (Steraloids Inc, Newport, RI, USA) at a final concentration of 1uM, in untreated tissue culture flasks (Nunc, Waltham, MA, USA). The cells were regularly incubated at 37°C and 5% CO_2_. The culture was fed every 5 days by raising the medium volume by 30% (with the feed medium containing the same concentrations of supplements as the original medium). The culture was passaged after every 10 days by first collecting and pelleting the neurospheres. The pellet was then re-suspended in 0.05% Trypsin with 0.53 mM EDTA and incubated for about 1.5 min at 37°C. 1ml of soybean trypsin inhibitor was next added, with trituration, to stop the trypsin activity and to dissociate the neurospheres into single cells. Cells were pelleted again, to remove the trypsin and inhibitor. Finally, cells to be used for serial passage were added to an appropriate volume of complete NS-A medium (described above), whereas cells to be used for transplantation were suspended in 1× PBS at 100,000 cells/μl and placed on ice.

### Transplantation

For transplantation, a single cell solution was prepared and stored on ice until transplantation. Each P2 mouse was anaesthetized by hypothermia and received a unilateral injection of 1 μl of cell suspension (100,000 hNPCs/μl PBS) into the right parietal cortex at a rate of ~0.25 μl/min using a 10-μl Hamilton microsyringe (Hamilton Company, Reno, NV, USA) fixed to a micromanipulator. After the injection, pups were allowed to recover before being returned to the dam.

### Electrophysiology

Each mouse was deeply anesthetized with isoflurane and decapitated. The brain was gently but quickly removed. Coronal brain slices (300 μm) were cut in an ice-cold cutting solution using a Vibratome (Leica VT1000 S, Leica Microsystems, Wetzlar, Germany). The cutting solution contained (in mM) 220 sucrose, 2.5 KCl, 1.25 NaH_2_PO_4_, 25 NaHCO_3_, 0.5 CaCl_2_, 7 MgCl_2_, and 10 D-glucose and was oxygenated with 95% O_2_–5% CO_2_ (pH 7.35~7.45 and osmolarity, 350~360 mOsm). Slices were incubated in extracellular solution for ≥1 h in a storage chamber at room temperature (RT, ~23°C) and were then transferred to a submerged chamber for recording. The extracellular solution contained (in mM) 125 NaCl, 2.5 KCl, 1.25 NaH_2_PO_4_, 26 NaHCO_3_, 2 CaCl_2_, 1.3 MgCl_2_, and 10 D-glucose and was constantly oxygenated (pH 7.35~7.45 and osmolarity 300~310 mOsm). All electrophysiological recordings were carried out at 30°C under visual guidance using an inverted microscope (Nikon Eclipse E600FN) equipped with infrared DIC optics and an × 40 water-immersion lens.

Conventional whole cell patch-clamp techniques were utilized [46]. Patch pipettes were pulled from Wiretrol II capillary glass (Drummond Scientific, Broomall, PA, USA) in a horizontal pipette puller (Model P-87 Flaming/Brown Micropipette puller, Sutter Instruments, Novato, CA, USA). Patch pipettes had resistances of 4~5 MΩ in the bath when filled with a recording electrode solution (in mM) containing 60 K-gluconate, 60 KCl, 8 NaCl, 10 HEPES, 2 MgATP, 0.3 Na_3_GTP, and 0.2 EGTA (pH 7.25 was adjusted with KOH, and osmolarity was 280~285 mOsm). We routinely added biocytin (0.2%) to the recording electrode solution to allow post-hoc morphological identification of the recorded cells. Using this intracellular solution, both spontaneous inhibitory/excitatory postsynaptic currents (sIPSCs and sEPSCs) were inward currents at a holding potential of-70 mV. The recordings were performed using a MultiClamp 700B amplifier (Axon Instruments). Data acquisition and analysis were performed using pClamp 10.1 software with a Digidata 1320A interface (Molecular Devices, Union City, CA). Signals were digitized at 10–20 kHz and analyzed off-line. Recordings were discarded if access resistance changed >10% during the experiment. Recording started 5–10 min after the whole cell patch was formed. The liquid junction potential was corrected using the Pipette Offset function of MultiClamp 700B before performing recording.

We identified GFP+ neurons using fluorescence microscopy and patched the cells using infrared differential interference contrast (IR-DIC) microscopy. Neurons were randomly chosen for recording and identified by morphology, electrophysiology and histology. Spontaneous IPSCs and sEPSCs were recorded from GFP+ cells and nearby host cells in layer V of cortical slices. Spontaneous IPSCs were recorded in the presence of *N*-methyl-d-aspartate (NMDA) and AMPA/kainate receptor antagonists, d-2-amino-5-phosphonopentanoic acid (d-AP5, 50 μM) and 2,3-dihydroxy-6-nitro-7-sulfamoyl-benzo quinoxaline-2,3-dione (NBQX, 10 μM), respectively. Spontaneous EPSCs were recorded in the presence of picrotoxin (PIC, 100 μM). The input resistance of cells was monitored by frequently applying a 100-ms hyperpolarizing voltage step of 10 mV from a holding potential of -70 mV.

### Immunohistochemistry

We performed immunofluorescence staining in sections after recording [47–48]. Slices with electrophysiologically identified biocytin-filled neurons were fixed in 4% paraformaldehyde and kept for 72 h at 4°C. To remove endogenous peroxidase, sections were quenched in 10% methanol and 3% H_2_O_2_ (in PBS) for 5 min. Sections were incubated for 1 hour at RT with both 2% normal donkey serum (NDS) and 1% bovine serum albumin (BSA) to block nonspecific binding and 0.5% Triton-100 in 1×PBS to permeate cell membranes. For labeling, sections were further incubated with primary antibodies in 2% NDS and 1% BSA and 0.5% Triton-100 in 1×PBS for 72 h at 4°C. After thorough rinsing, all sections were incubated with secondary antibodies at RT for 2.5 h. The primary antibodies were rabbit anti-parvalbumin (PV) antibody (EMD Millipore, Billerica, MA, USA; diluted at 1:2000), goat anti-calretinin (CR) antibody (EMD Millipore; diluted at 1:2500), mouse anti-somatostatin (SS) antibody (GeneTex, Irvine, CA, USA; diluted at 1:100), and mouse anti-human nuclei (hNuc) monoclonal antibody (EMD Millipore, 1:200). The secondary antibodies were Alexa Fluor 350 donkey anti-rabbit (for PV), goat (for CR), and mouse (for SS) immunoglobulin G (IgG), Alexa Fluor 594 streptavidin for biocytin, and Alexa Fluor 594 donkey anti-mouse IgG for hNuc (Invitrogen, Carlsbad, CA, USA; diluted at 1:400). After staining, slices were mounted on glass slides in fluoromount aqueous mounting medium (Sigma), coverslipped and sealed with clear nail polish for imaging. Sections were examined with an Olympus IX81-DSU Spinning Disk Confocal Microscope (Olympus America, Melville, NY, USA). Serial images from each section were acquired with a z step of 0.5 μm and an image size of 672×512 pixels. Z-axis image stacks were created from serial images. Cell counts were performed from stacked images using ImageJ software version 1.37V (Wyne Rasband, National Institutes of Health).

### Analysis

Action potential (AP) threshold was obtained from a first derivative plot where the dV/dt abruptly increased (5 V s^-1^). AP amplitude was measured from the threshold to peak. Spike widths were measured at half amplitude of APs. The membrane time constant was computed by the monoexponential curve fitting of voltage responses to hyperpolarizing current pulses. The slope (in Hz/nA) of the linear regression was determined by the relationship between injected current intensity and firing rates (*f*–*I*); AP adaptation was defined as the ratio of the last interspike interval (ISI) to the first ISI of APs. Analysis of synaptic currents sEPSCs and sIPSCs was based on 5 min of continuous recording from each cell to obtain averaged data. Currents were analyzed using the MiniAnalysis Program (Synaptosoft, Leonia, NJ). The instantaneous amplitude and frequency were acquired to obtain mean values. The threshold for IPSC and EPSC detection was 6 pA, and the automatic detection was verified post hoc by visual inspection.

### Chemicals

D-AP5, NBQX, PIC and biocytin were purchased from Sigma. Suppliers of primary and secondary antibodies were described in the preceding text.

## Results

### Identification of different types of whole cell recorded neurons

Serial 300 μm thick coronal slices of cortex were cut and there were 2–4 slices with GFP+ cells per animal. GFP+ cells were found scattered in an area extending 0.6–1.0 mm in the rostro-caudal direction and 0.5–1.1 mm in the medio-lateral direction, primarily in cortical layers III–VI. The recordings of GFP+ cells were performed in layer V to compare with data obtained from host cells.

**Fig 1:**
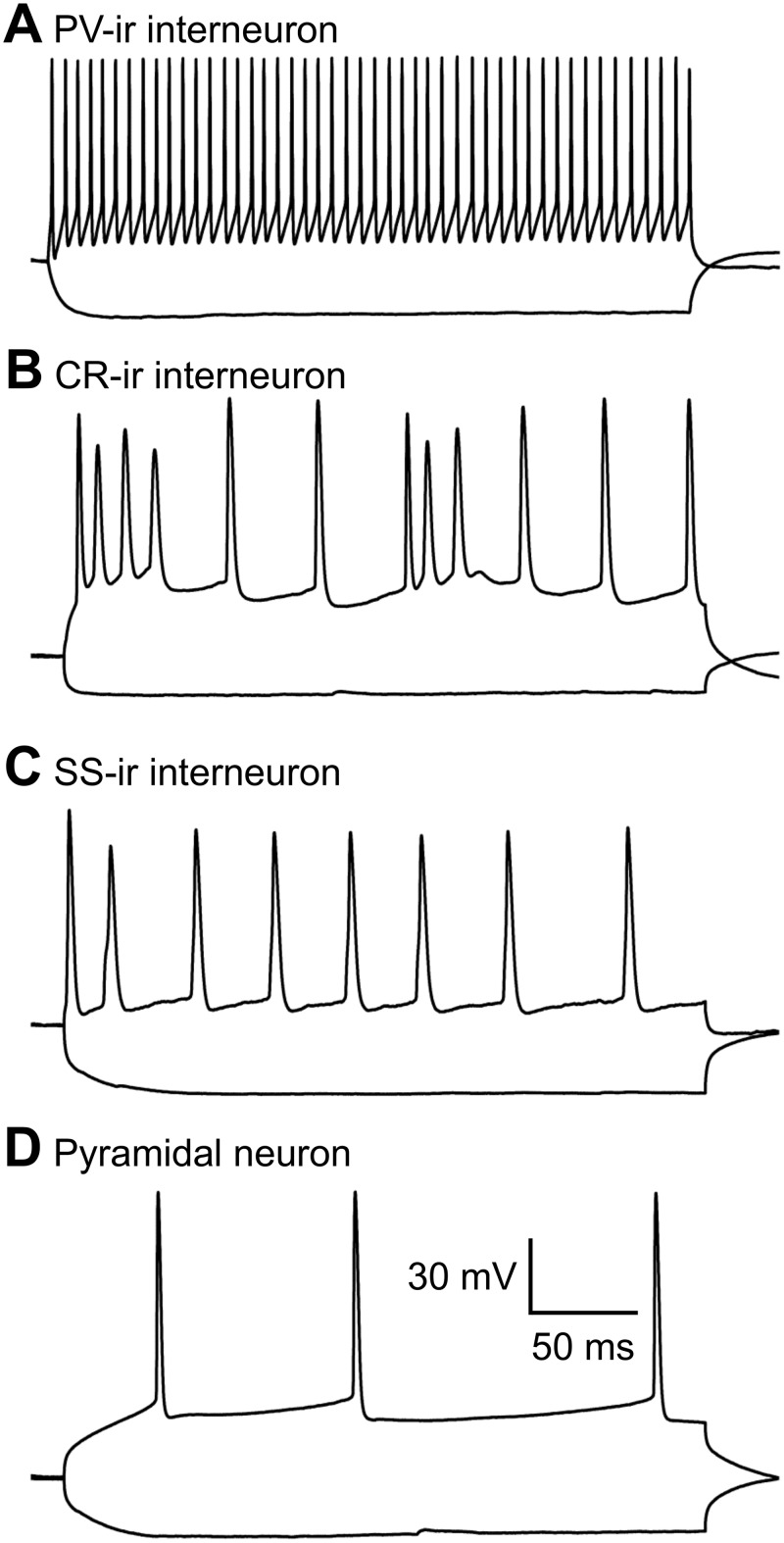
Firing patterns of four different types of hNPCs. Whole-cell current-clamp responses to injection of depolarizing (300 ms, +250 pA) and hyperpolarizing (300 ms, −250 pA) current pulses. Depolarizing current induced firing activity with different frequencies and patterns in 4 subtypes of hNPCs. In response to depolarizing current, PV-ir cells fired at high frequency without any adaptation (A); CR-ir cells fired with an irregular firing pattern (B); SS-ir cells fired at lower frequency with adaptation (C), and pyramidal neurons fired at the lowest frequency with obvious adaptation. The calibration bar in D also applies to A, B and C.

**Fig 2:**
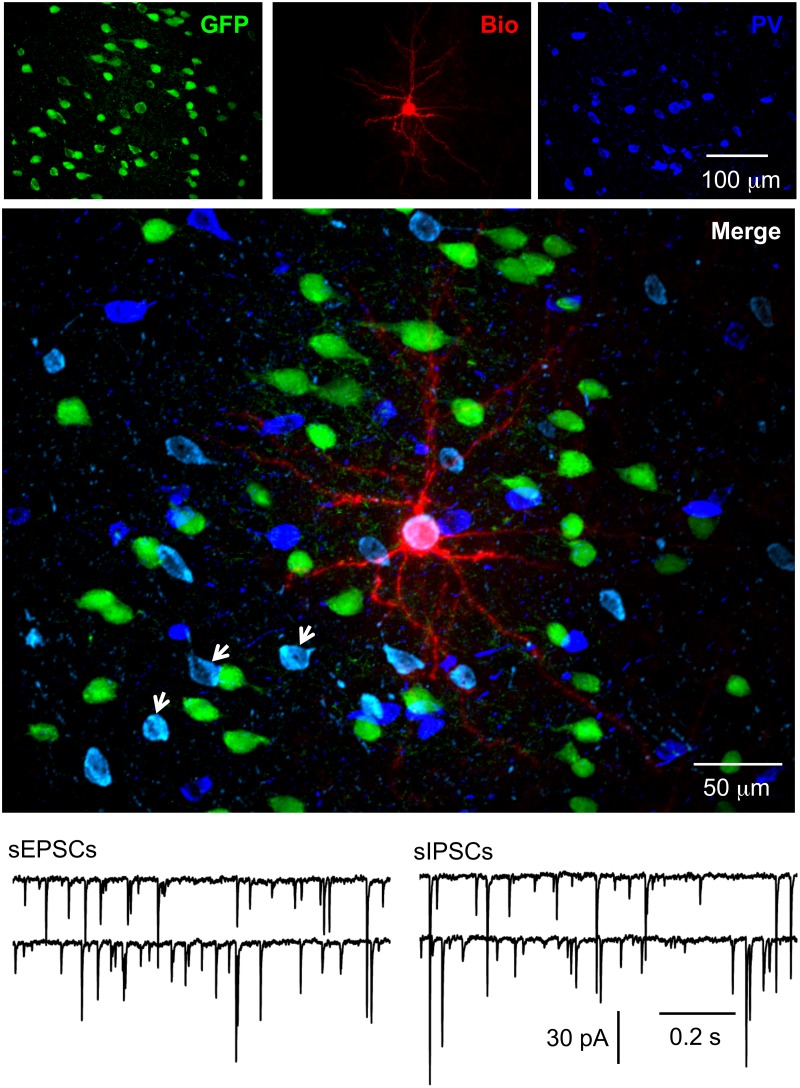
Functionally integrated PV-ir hNPCs. The representative hNPC was recorded in the whole-cell configuration. After recording, the slice was fixed and further processed for PV staining. The electrophysiologically identified hNPC proved to be a PV-ir interneuron. Spontaneous IPSCs, as shown in this figure, were observed in this neuron in the presence of NBQX and d-AP5, and after 20 min washout, sEPSCs were observed in the presence of PIC (not shown). To exclude the possible complications of the addition of antagonists, representative traces for spontaneous EPSCs from another PV-ir hNPC were shown in the figure in the presence of PIC. Series images from each section with GFP+ hNPCs and biocytin staining were acquired with a z-step of 0.5 μm and stacked along the Z-axis. In the merged image, the neuron in white (GFP+Biocytin+PV) was a grafted hNPC that was recorded in whole cell mode and stained with PV; neurons in cyan (GFP+PV) were PV-ir hNPCs that were not recorded (arrows); neurons in green (GFP) were PV negative hNPCs; neurons in blue (PV) were PV-ir interneurons that were from host mouse cells. Note that anti-PV reacted with both human and mouse neurons; however, mouse PV-ir interneurons were all GFP negative.

**Fig 3:**
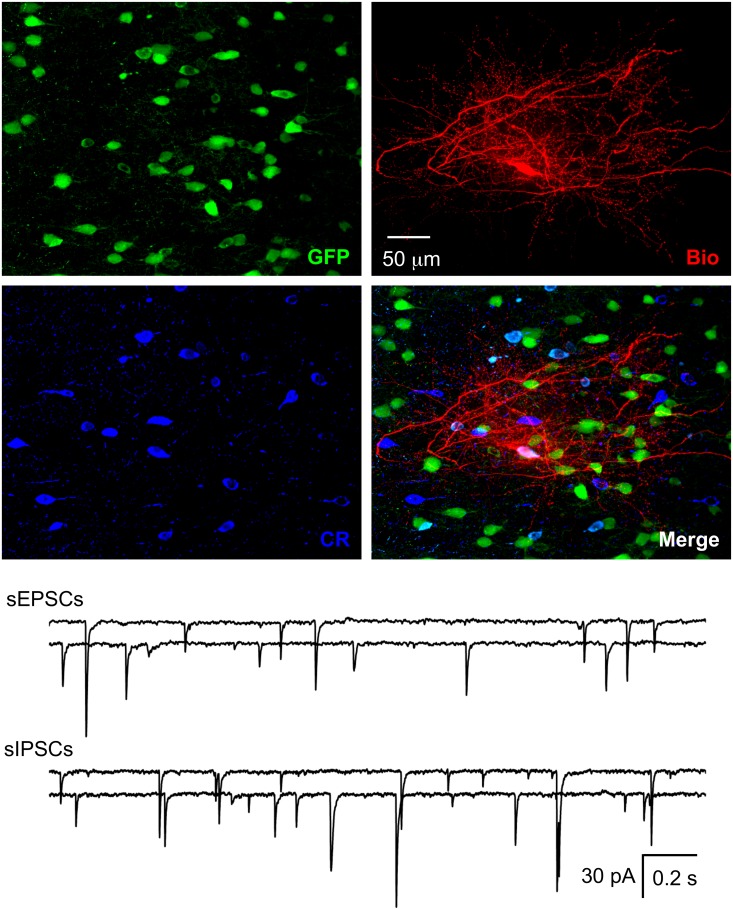
Functional integration of CR-ir hNPCs. The section with the recorded hNPC was further processed with anti-CR and the recorded hNPC was found to be a CR-ir interneuron. Spontaneous IPSCs and EPSCs were found in CR-ir hNPCs. Representative traces for sIPSCs and sEPSCs from two CR-ir interneurons are shown.

**Fig 4:**
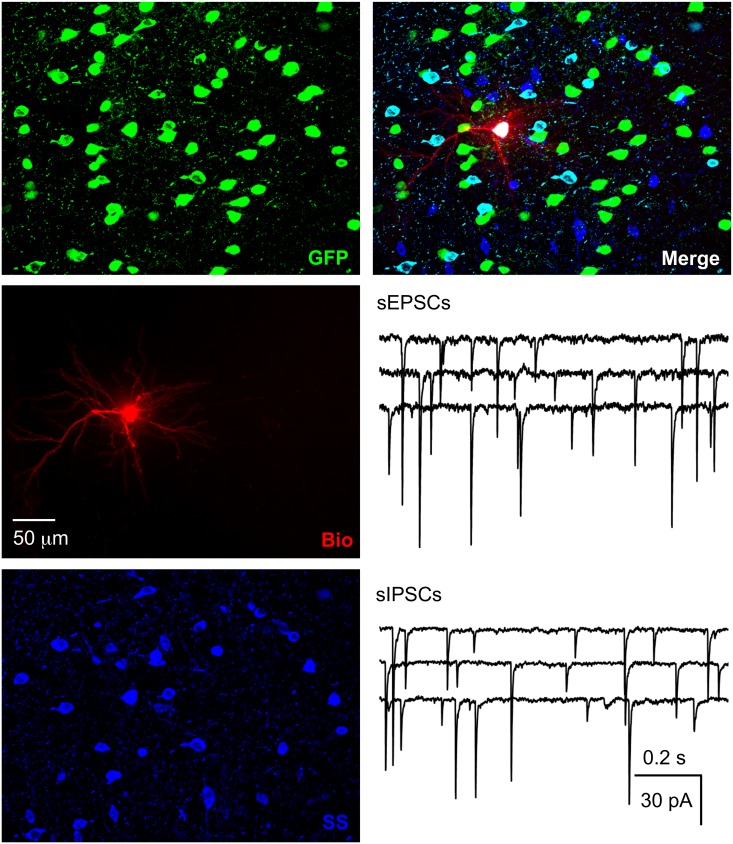
Functional integration of SS-ir hNPCs. The section with the recorded hNPC was further processed with anti-SS and the recorded hNPC was a SS-ir interneuron. Spontaneous IPSCs and EPSCs were recorded in SS-ir hNPCs. Traces for sIPSCs and sEPSCs were recorded from two SS-ir interneurons.

**Fig 5:**
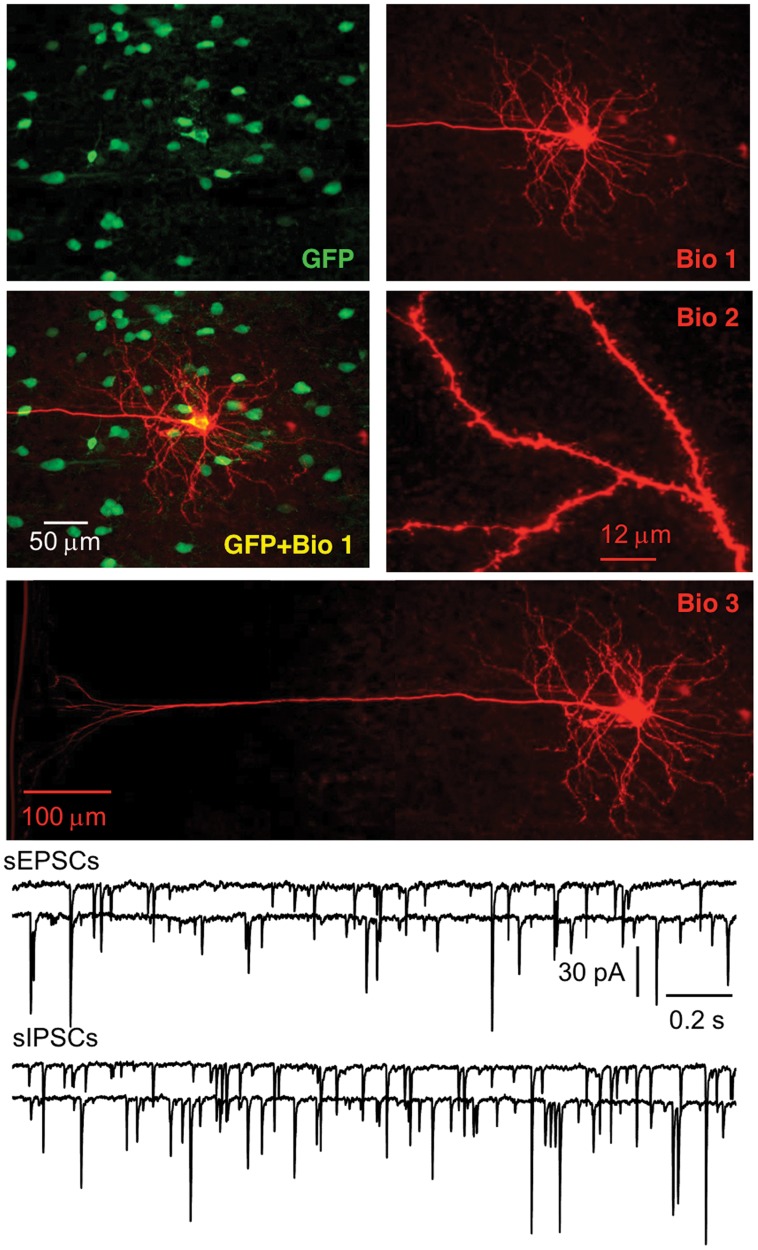
Physiological integration of hNPC-derived pyramidal neurons in the cortex. Human NPCs developed into neurons with a typical pyramidal soma, a long and thick apical trunk, and dendritic spines. Spontaneous IPSCs and EPSCs are shown from two pyramidal neurons.

There are various types of interneurons, but each type has a unique combination of firing patterns and molecular markers [37, 47–48], which enabled us to identify these GFP+ cells. For firing patterns of GFP+ cells in response to depolarizing current, putative PV-ir cells displayed high frequency repetitive discharges without adaptation, putative CR-ir cells fired an initial spike burst followed by irregularly spaced APs, and putative SS-ir cells exhibited lower frequency firing (note that it was lower than putative PV-ir cells, but higher than pyramidal cells) with adaptation (Fig. 1). To further identify the type of biocytin-filled GFP+ cell, sections with electrophysiologically identified GFP+ putative PV-, CR- or SS-ir cells were incubated with anti-PV, anti-CR or anti-SS antibody, respectively. At 8 W after transplantation, we recorded from 30, 15 and 17 cells (62 total) that were electrophysiologically identified as putative PV-, CR- and SS-ir interneurons, respectively. Of those cells, 25, 12 and 15 (50 total) were confirmed to be PV-, CR- and SS-ir interneurons, respectively, with biocytin labelling and post-hoc immunohistochemistry (Figs. 2–4). Twelve of 62 electrophysiologically identified interneurons were not further identified histologically. Eleven of 11 electrophysiologically and morphologically identified pyramidal neurons were recorded and all were included for analysis. The 12 electrophysiologically identified interneurons that were not verified by histology, and 10 recorded cells that were not identified by either electrophysiology or histology (they were neither pyramidal neurons nor interneurons with staining of PV, CR or SS) were excluded from this study. GFP+ PV-, SS- and CR-ir interneurons lacked long, thick apical dendrite (Figs. 2–4). Recorded GFP+ pyramidal cells were characterized by their pyramidal soma, a single long, thick apical dendrite (Fig. 5), and slower firing rates with obvious frequency adaptation (Fig. 1) that were easily distinguished from recorded GFP+ interneurons.

### Functional integration of different types of GFP+ neurons

We examined sEPSCs and sIPSCs from GFP+ interneurons and pyramidal neurons. At 8 W after transplantation, sEPSCs and sIPSCs were detected in GFP+ PV-, CR- and SS-ir interneurons; they were also present in GFP+ pyramidal cells (Figs. 2–5, Table 1). These results demonstrate that GFP+, hNPC-derived interneurons and pyramidal neurons received both excitatory and inhibitory inputs, indicating that they were functional and able to interact with other neurons in the neuronal network.

**Table 1:** Intrinsic membrane properties and synaptic currents of different types of neurons.

	PV		CR		SS		Pyramids	
	GFP	Host	GFP	Host	GFP	Host	GFP	Host
***Membrane properties***								
**Rest potential (mV)**	-73.7±1.2	-74.2±1.5	-70.4±1.4	-71.1±1.5	-74.5±1.6	-73.7±1.4	-74.9±1.5	-75.3±1.7
**AP threshold (mV)**	-48.4±0.9	-47.6±1.1	-51.5±1.7	-49.8±1.5	-47.9±1.1	-48.3±1.2	-51.6±1.3	-50.2±1.2
**AP amplitude (mV)**	71.3±1.0	72.4±1.3	54.1±2.0	56.9±1.7	69.9±1.4	70.7±1.6	69.5±1.1	70.3±1.4
**AP half-width (msec)**	0.45±0.03	0.43±0.05	0.86±0.10	0.81±0.12	0.64±0.08	0.62±0.06	1.22±0.09	1.19±0.10
**R_input_ (MΩ)**	170.5±13.4	165.1±14.8	275.6±29.3	281.4±31.5	183.2±21.2	179.9±20.6	194.5±15.5	183.3±14.7
**Time constant (msec)**	14.4±1.2	13.8±1.5	19.7±1.8	18.6±2.1	15.8±1.7	15.3±1.9	19.1±1.3	19.3±1.4
***f-I* slope (Hz/nA)**	324.4±14.5	338.5±15.5	142.4±16.1	149.6±17.5	101.2±12.4	108.7±13.7	54.8±7.9	59.5±7.3
**AP adaptation**	0.97±0.07	0.99±0.08	5.63±0.85	5.48±0.91	2.74±0.47	2.63±0.40	2.13±0.24	2.05±0.22
**N**	25	11	12	10	13	11	11	12
***sEPSCs***								
**Frequency (Hz)**	9.03±0.55	9.34±0.73	2.31±0.14	2.40±0.18	3.49±0.30	3.68±0.37	6.08±0.42	5.47±0.53
**Amplitude (pA)**	34.6±1.5	31.7±2.2	24.0±1.8	24.6±2.1	25.8±1.7	26.2±2.1	31.9±2.4	30.7±2.2
**Rise time (msec)**	1.42±0.09	1.21±0.10	1.47±0.11	1.27±0.12	1.40±0.07	1.22±0.09	1.67±0.12	1.55±0.16
**τ(msec)**	9.56±0.71	8.58±0.82	9.67±0.80	8.34±0.85	9.09±0.85	8.54±0.83	10.25±0.82	9.18±0.97
**N**	13	5	6	5	6	6	5	6
***sIPSCs***								
**Frequency (Hz)**	7.64±0.48	6.69±0.57	2.64±0.21	2.77±0.23	2.77±0.19	2.96±0.25	13.12±1.14	12.78±1.18
**Amplitude (pA)**	32.2±2.1	28.9±2.5	23.6±1.8	24.4±2.2	24.9±1.7	25.1±2.0	36.4±2.6	34.7±2.8
**Rise time (msec)**	1.82±0.10	2.14±0.16	1.77±0.12	1.91±0.18	1.85±0.14	2.08±0.15	1.93±0.14	1.99±0.17
**τ(msec)**	14.27±1.02	16.74±1.42	14.06±1.13	16.75±1.44	14.94±1.16	16.65±1.34	13.47±0.80	12.97±1.16
**N**	12	6	6	5	7	5	6	6

PV, CR and SS are PV-, CR-, and SS-ir interneurons, respectively; AP, action potential; R_input_, input resistance; *f*-*I* slope, slope of the relationship between injected current intensity and firing rate;τ, decay time constant; rise time, 10–90% rise time.

The intrinsic electrophysiological properties of different types of GFP+ cells were not significantly different from host NSG mouse neurons of the same type (Table 1). The frequency and amplitude of sEPSCs and sIPSCs of grafted GFP+ neurons were also comparable to host neurons (Table 1).

### Co-localization of GFP with neuropeptides or HNuc

We performed double or triple staining in sections after recording at 8 W after transplantation. We analyzed the co-localization of GFP with three neuropeptides (PV, CR and SS) and human nuclear antigen (hNuc). Because anti-PV, CR and SS antibody can react with PV, CR and SS from both human and mouse tissue, we counted the number of cells with co-localization of GFP and PV, CR or SS that represented the number of implanted hNPCs with one of three specific markers of interneurons (Figs. 2–4). We cut serial coronal slices of cortex and 2–4 slices per animal had GFP+ cells. All GFP+ cells from slices were counted, and we found that the average percentage of surviving GFP+ cells was 1.21 ± 0.16% (averaged 1,214 ± 156 surviving GFP+ cells per animal from 100,000 implanted cells, Figs .2–5; n = 23 NSG mice from 4 pregnant mice) at 8 W after transplantation. In a few cases, not all slices were collected because of failure of the cutting procedure, and GFP+ cells could not be exhaustively counted. Therefore, our numbers may represent an underestimate of total surviving transplanted cells. The proportion of PV-, CR- and SS-ir cells among GFP+ cells was 35.5 ± 3.8% (averaged 106 ± 12 PV-ir cells/298 ± 32 GFP+ cells, n = 30 sections), 15.7 ± 1.7% (averaged 46 ± 5 CR-ir cells/288 ± 43 GFP+ cells, n = 15 sections) and 17.1 ± 1.8% (averaged 49 ± 6 SS-ir cells/282 ± 43 GFP+ cells, n = 17 sections), respectively. Thus, the three subtypes of interneurons accounted for 67.9% of the GFP+ cells. Around 15% of GFP+ cells could be identified as putative pyramidal neurons by their unique morphology and another ~ 15% GFP+ cells were not identified by histology and morphology. The maximum area occupied by GFP+ cells in the cortical sections was 0.76 ± 0.04 mm^2^ (n = 23 mice).

**Fig 6:**
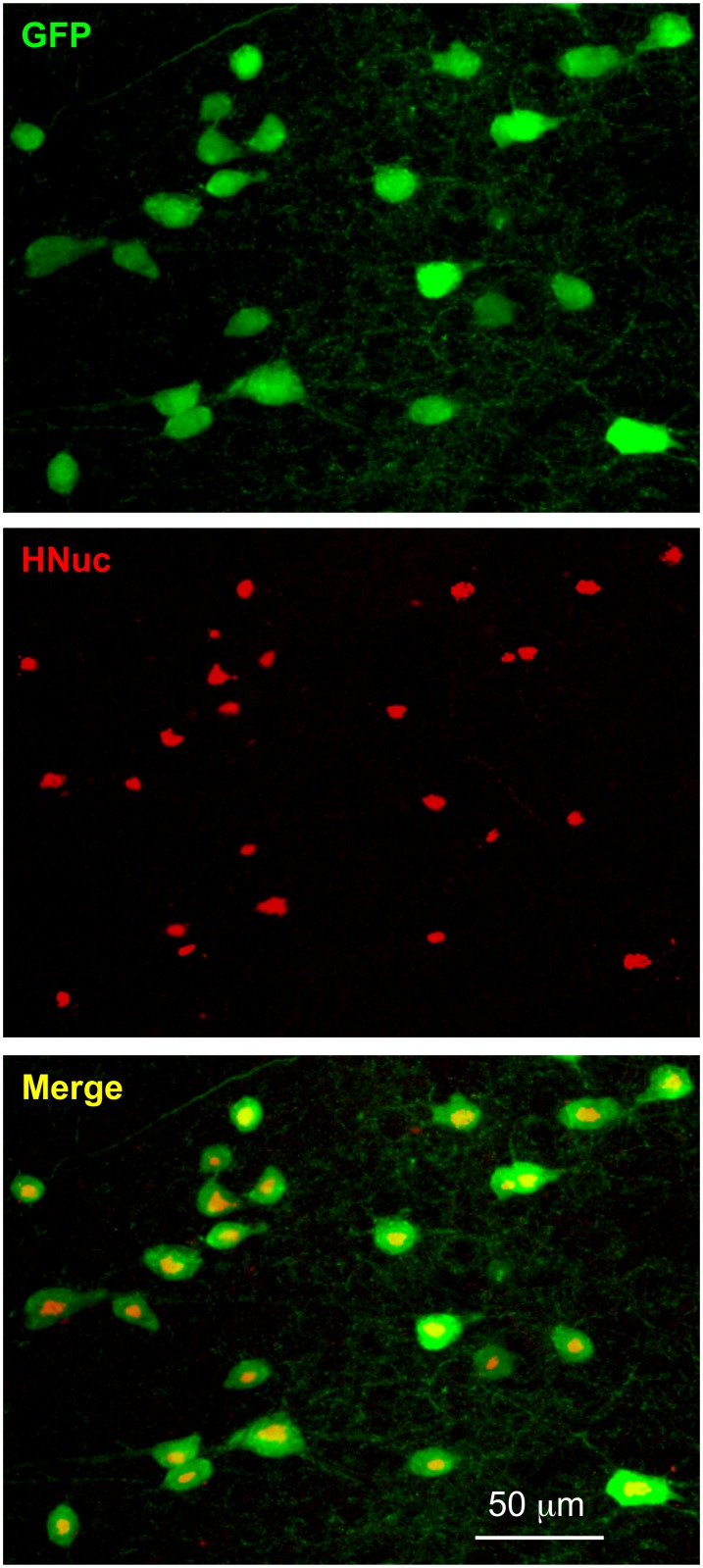
Immunoreactivity of hNPCs with an antibody against hNuc. All putative hNPCs (GFP+) were hNuc+ cells at 8 W after transplantation (see merged image). No mouse cells were hNuc+.

At 8 W after transplantation, all the GFP+ cells were positive for anti-HNuc, a specific antibody for human nuclei that serves as a histological marker for identifying human cells in xenograft models (Fig. 6). We confirmed that no immunoreactivity to HNuc was observed in GFP- cells in mice with or without transplantation (all n = 10 sections from 2 mice), suggesting that GFP+ cells originated from hNPCs.

## Discussion

The present study demonstrates that hNPCs at 8 W after transplantation can develop into different classes of phenotypically-identified neurons: PV-, CR- and SS-positive inhibitory interneurons and excitatory pyramidal neurons that are able to fire action potentials and functionally integrate into existing networks in the cortex of immunodeficient NSG mice.

Stem cell survival, migration, differentiation and improvement of neurologic function could depend on many factors including age and region of tissue for stem cell acquisition, differentiation in vitro before transplantation, number of transplanted cells, adjuvants for transplantation, age of host at the time of transplantation, anatomical site of transplantation, immune response, treatments (e.g., use of immunosuppression or immunodeficient animals), animal model of human diseases or extent and type of host injury (e.g., the MPTP-lesioned mouse model of Parkinson’s disease, the pilocarpine-induced mouse model of temporal lobe epilepsy and ischemic rat cerebral cortex), and access to the vestigial migratory stream (e.g., the subventricular zone of the lateral ventricles and the subgranular zone of the dentate gyrus in the hippocampus) [12, 14, 18, 24, 32, 49–55]. With this many variables, research for stem cell transplantation can be very complex and the results can vary greatly between different protocols.

Our present results show that hNPCs can survive at least 8 W (the furthest point examined in this study) when transplanted in P2 immunodeficient NSG mice. Prior studies have reported survival of human neural stem cells throughout the mouse brain for at least 7 months after transplantation into neonatal mouse lateral ventricle [56]. Transplanted animal stem cells can survive and remain functional for longer than 1 year [17] and even for the life of the host animal [57]. Many factors play important roles in survivability of transplanted cells. In general, survival of transplanted cells is host age-dependent, with longer survival of transplants in younger, rather than older, host brains [58]. The underlying mechanism is uncertain, but the expression of age-dependent host factors including neurotrophic factors and cell-adhesion molecules may contribute the differences in survival [59]. Transplanted cells are also likely recognized as foreign and the majority of transplant studies have incorporated immunosuppressive therapy or were performed in immunodeficient animals to mitigate graft rejection and enhance survivability, particularly in xenografts [54, 60]; although graft rejection and graft survival in both non-immunosuppressed and immunosuppressed recipients have been observed [16, 61]. Survival is also dependent on the time that the transplanted cells had spent differentiating in vitro; presumably, mature neurons survive transplantation more poorly than do immature neurons. Grafts of human fetal neural progenitor cells that had been expanded for a longer time in vitro exhibited poorer survival rates after transplantation into neonatal rat hippocampus [62]. We transplanted hNPCs into P2 immunodeficient NSG mice in the present study. Our donor cells were somewhat differentiated in that they produced only neurons and no glia. However, the fact that they were able to produce both GABAergic interneurons and glutamatergic pyramidal neurons suggests that they were early in their ontogenetic development as neural precursor cells, or that our donor population contained a mixture of committed interneuron precursors and pyramidal cell precursors. We found that only 1.21% of GFP+ cells survived for 8 W, the furthest point examined in this study. The relatively low survival rate may be due, at least in part, to cortex as an unfavorable anatomical site for transplantation. Currently we have no data on survival of hNPC transplants beyond 8 W. We did observe a significant decline in the number of human cells before 4 weeks after transplantation and then the decline started to slow down (unpublished data). The significant decline may be due to graft rejection that develops gradually and progresses further when transplanted stem cells differentiate into different types of neurons and express human specific cell surface markers, but fail to establish functional integration with the host tissue.

Previous studies have shown that transplanted cells respond to local signals and differentiate into different functional types of neurons typical for a specific anatomical region/microenvironment [14, 49, 52, 63]. Transplanted human neural progenitor cells can develop into neurons with expression of calbindin in the Purkinje cell layer of the rat cerebellum [16], tyrosine hydroxylase positive neurons in the striatum of MPTP mice and dopaminergic neurons in the striatum of the 6-hydroxydopamine-lesion rat model of Parkinson’s disease [53], and PV- and SS-positive neurons in cortex [20]. Fetal stem cells from animals can develop into dopaminergic neurons in the host rat neostriatum [64] and excitatory pyramidal neurons and inhibitory interneurons that functionally integrate into the host rat neocortex and hippocampus [11, 63]. We identified three types of GFP+ interneurons (PV-, CR- and SS-positive) and excitatory GFP+ pyramidal neurons in neocortex that accounted for most of the GFP+ cells. Interestingly, the majority of GFP+ neurons (67.9%) developed into interneurons and only a small percentage developed into pyramidal neurons (around 15%) in neocortex, in contrast to a higher percentage of pyramidal neurons (70–80% of neurons) and lower percentage of interneurons (20–30%) in host neocortex. It is possible that the host microenvironment favors the development of interneurons over pyramidal neurons. This could be very important, because many neurological diseases develop due to reduced inhibition, and replacing lost or malfunctioning inhibitory interneurons could help cure these disorders. Alternatively, the ability to produce excitatory pyramidal neurons may prove beneficial in other clinical settings.

It is noteworthy that previous studies have shown the development of animal stem cells into functional pyramidal neurons [63, 65–66]. Human fetal neural stem cells have been shown to generate neurons, although not physiologically identified pyramidal neurons [13–18]. Our present data show that hNPCs can generate pyramidal cells with characteristic somatodendritic morphologies and physiological profiles that functionally integrate into the host neural network. However, the presence of EPSCs and IPSCs in the post-synaptic cells does not tell us the identity of the pre-synaptic cell. It is possible that the majority of PSCs are coming from other hNPC-derived neurons rather than host neurons. However, we think that this is unlikely for the following reason. In this study, hNPCs produced relatively few excitatory pyramidal cells (15%). If all or most of the PSCs that we recorded in hNPC-derived postsynaptic neurons actually originated from other hNPC-derived presynaptic neurons, there should have been a great preponderance of IPSCs compared to EPSCs. We found just the opposite; that the frequency of EPSCs and IPSCs was not different in hNPC-derived neurons compared to host neurons of the same type.

## Conclusions

Although limitations of human transplantation strategies include restricted tissue supply, variability in response, and the rejection of transplanted cells by the host's immune system, stem cell transplantation remains a promising method to treat neurological diseases through replacement of lost neurons, enhancement of intrinsic neuroplasticity of neurons, and/or facilitation of neuronal migration from other regions to the site of injury. This study demonstrates that hNPCs are able to survive for an extended period of time and that the great majority of transplanted cells can develop into interneurons that could be important in the treatment of neurological diseases associated with the loss of inhibitory interneurons, such as epilepsy.
